# Macroscopic and Macromolecular Specificity of Alkylphenol Anesthetics for Neuronal Substrates

**DOI:** 10.1038/srep09695

**Published:** 2015-04-08

**Authors:** Brian P. Weiser, Michael A. Hall, Nathan L. Weinbren, Kellie A. Woll, William P. Dailey, Maryellen F. Eckenhoff, Roderic G. Eckenhoff

**Affiliations:** 1 Department of Anesthesiology and Critical Care, University of Pennsylvania, Philadelphia, PA; 2 Department of Pharmacology, University of Pennsylvania Perelman School of Medicine, Philadelphia, PA; 3 Department of Chemistry, University of Pennsylvania, Philadelphia, PA

## Abstract

We used a photoactive general anesthetic called *meta*-azi-propofol (AziP*m*) to test the selectivity and specificity of alkylphenol anesthetic binding in mammalian brain. Photolabeling of rat brain sections with [^3^H]AziP*m* revealed widespread but heterogeneous ligand distribution, with [^3^H]AziP*m* preferentially binding to synapse-dense areas compared to areas composed largely of cell bodies or myelin. With [^3^H]AziP*m* and propofol, we determined that alkylphenol general anesthetics bind selectively and specifically to multiple synaptic protein targets. In contrast, the alkylphenol anesthetics do not bind to specific sites on abundant phospholipids or cholesterol, although [^3^H]AziP*m* shows selectivity for photolabeling phosphatidylethanolamines. Together, our experiments suggest that alkylphenol anesthetic substrates are widespread in number and distribution, similar to those of volatile general anesthetics, and that multi-target mechanisms likely underlie their pharmacology.

Propofol (2,6-diisopropylphenol) is one of the most commonly used intravenous general anesthetics. However, in addition to propofol, other molecules of the same alkylphenol chemotype are efficacious and potent general anesthetics^1^,^2^,^3^,^4^, and some of these are being investigated for clinical use^5^,^6^,^7^. Despite this, the pharmacologic mechanisms that underlie alkylphenol anesthesia remain unclear across molecular, cellular, and neural systems levels. Because the alkylphenols likely cause hypnosis through conserved mechanisms, continuing to characterize the pharmacology of this chemotype should ultimately improve their use and development.

Alkylphenol general anesthetics are approximately two orders of magnitude more potent than volatile anesthetics. This could be due to higher affinities of the alkylphenols for drug targets and/or higher efficacies for modulating the function of critical substrates. One implication of higher affinity interactions is more selective binding to targets. To test the selectivity of binding, we characterized the macroscopic distribution of an alkylphenol anesthetic in its presumed target, the brain, and compared our results to that of the volatile anesthetic halothane^8^,^9^. For these and other experiments, we used a radiolabeled compound called *meta*-azi-propofol (AziP*m*). AziP*m* has similar potency to propofol *in vivo*^3^,^10^; however, AziP*m* can serve as a photoaffinity label, which allows for covalent attachment of the radioactive probe to its equilibrium binding sites for subsequent characterization. To interpret the macroscopic distribution in brain, we also investigated the selectivity of alkylphenol binding to protein and lipid macromolecules, as well as the specificity (i.e., saturability) of ligand binding to substrates.

## Results and Discussion

### Brain Section Photolabeling

**Figure 1:**
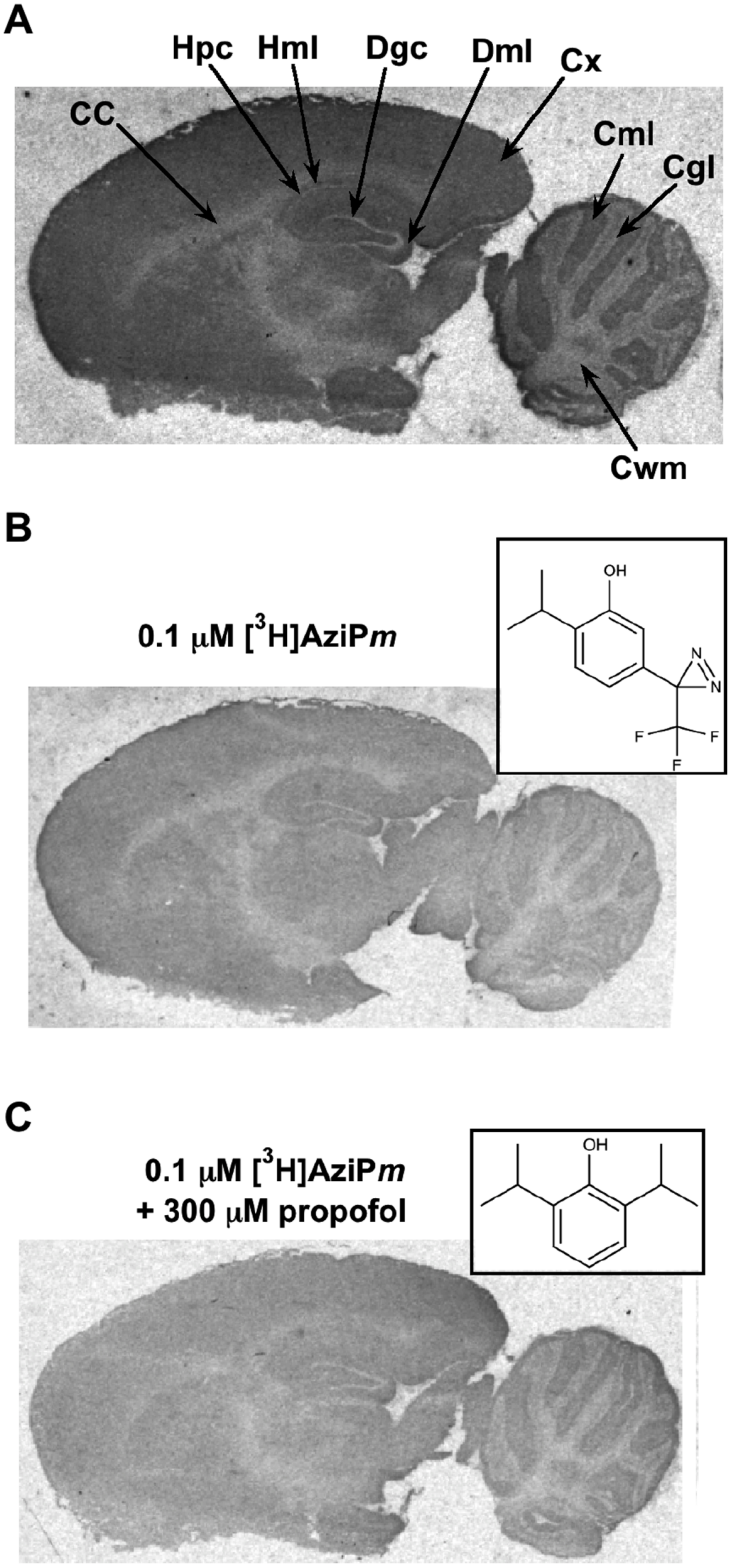
(A) Heavily contrasted autoradiograph of a sagittal brain section photolabeled with 0.1 μM [^3^H]AziP*m*. Regions of interest are indicated: CC, corpus callosum; Hpc, hippocampal pyramidal cell layer; Hml, hippocampal molecular layer; Dgc, dentate granule cell layer; Dml, dentate molecular layer; Cx, cortex; Cml, cerebellar molecular layer; Cgl, cerebellar granular cell layer; Cwm, cerebellar white matter. (B) Brain section photolabeled with 0.1 μM [^3^H]AziP*m* or (C) [^3^H]AziP*m* + 300 μM propofol. The insets depict (B) AziP*m* and (C) propofol. The sections in (B) and (C) were exposed to the same film and contrasted identically after development, and hence accurately portray relative levels of [^3^H]AziP*m* binding.

Brain sections equilibrated and photolabeled with 0.1 μM [^3^H]AziP*m* were exposed to x-ray film for autoradiography. We quantified binding to nine distinct brain regions (Fig. 1A, Fig. 1B, and Table 1). AziP*m* binding was widespread but heterogeneous, with the most heavily labeled regions (cortex and dentate molecular layer) approximately twice as intense as the least labeled (cerebellar white matter). We compared the selectivity of alkylphenol binding to that of halothane (Table 1). Overall, the relative selectivity of these chemically distinct anesthetics for each brain region was similar, and the largest differences were less than two-fold (Table 1).

The quantified brain regions can be combined into three compositions: (1) synapse-dense, (2) primarily cell bodies, and (3) primarily white matter (Table 2)^8^. Comparing combined data from each compositional class revealed that AziP*m* preferentially binds synapse-dense regions as compared to cell bodies or white matter (Table 2). Preferential binding suggests a concentration of substrates, or else generally higher affinity substrates, for alkylphenol anesthetics in these protein-rich areas. Maximal binding to synapse-dense regions was also seen for halothane, but halothane bound to white matter more strongly than did AziP*m*^8^. Although binding does not necessarily imply functional association with the anesthetic endpoint, concentrated binding at synapses for both drugs is consistent with the consensus that the major effect of general anesthetics is on synaptic transmission rather than neuronal conduction^11^.

Pharmacological specificity of alkylphenol sites on neuronal substrates could be indicated by inhibition of photolabeling by propofol. Therefore, we photolabeled brain sections with 0.1 μM [^3^H]AziP*m* while co-incubating with increasing concentrations of propofol (3–300 μM). We did not detect any significant change in total binding in any brain region, even with propofol concentrations 3000 fold higher than [^3^H]AziP*m* (Table 1 and Fig. 1C). We hypothesized that a high non-specific component of binding to lipid reduced the ability to detect saturable binding to protein in the whole brain section preparation, so we separately analyzed the specificity of alkylphenol anesthetic binding to both protein and lipid.

### Protein Photolabeling

**Figure 2:**
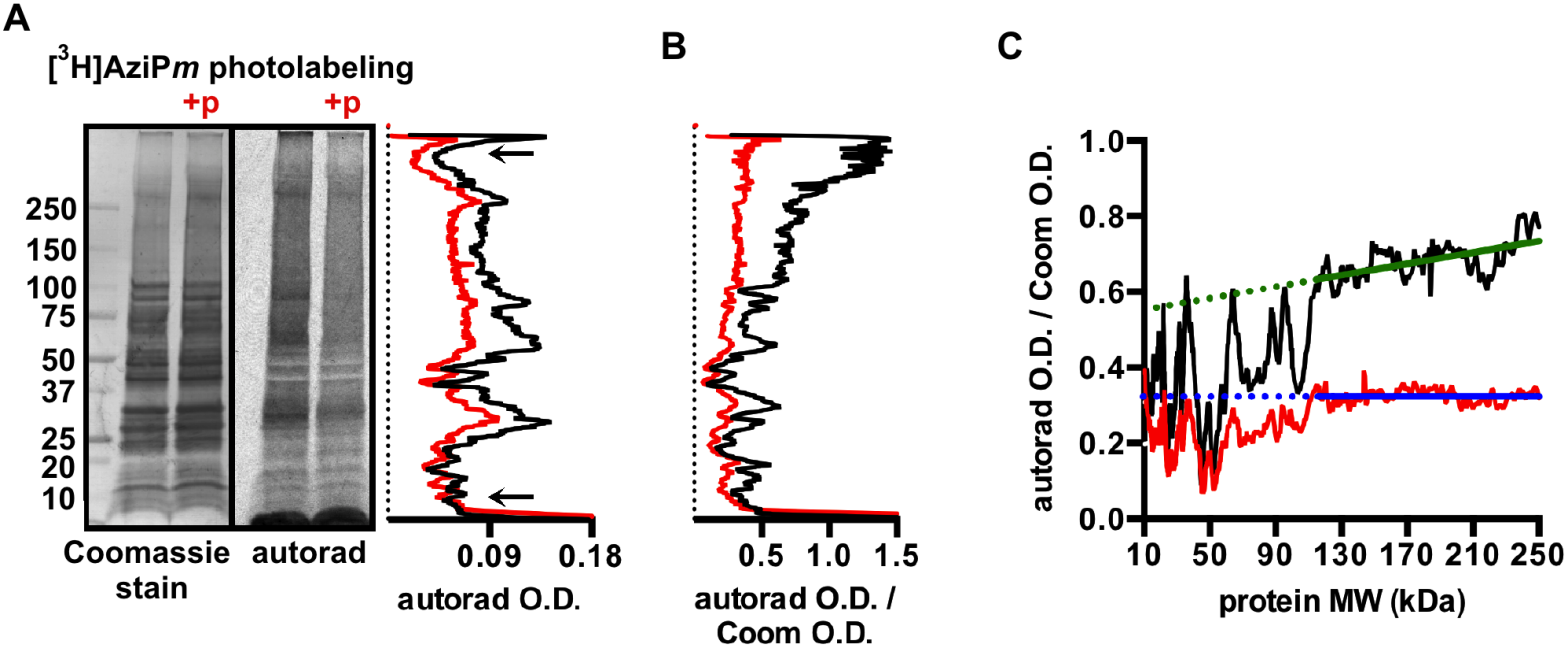
(A) Coomassie-stained PVDF membrane and corresponding autoradiograph of synaptosomal protein photolabeled with 4 μM [^3^H]AziP*m* or 4 μM [^3^H]AziP*m* + 400 μM propofol, with the latter indicated as +p. On the right, the optical density (O.D.) quantification of the lanes are shown, with [^3^H]AziP*m* shown in black, and [^3^H]AziP*m* + propofol shown in red. Between the arrows in the lanes, propofol inhibited [^3^H]AziP*m* photolabeling by an average of 31%. (B) Autoradiograph O.D. normalized to Coomassie O.D. for the membrane and autoradiograph shown in (A). Data for [^3^H]AziP*m* and [^3^H]AziP*m* + propofol are again shown in black and red, respectively. (C) The 10–250 kDa portion of the [^3^H]AziP*m* (black) and [^3^H]AziP*m* + propofol (red) traces from (B) are shown. Linear regression was used to fit lines (shown in green and blue) through the data between 115–250 kDa, and the traces were extended with the dashed line to 10 kDa. R^2^ was 0.38 and 0.00 for the green and blue fits, respectively.

To investigate [^3^H]AziP*m* binding to protein, we photolabeled isolated rat synaptosomes with and without propofol. The synaptosome fraction should contain the synaptic substrates that were strongly photolabeled in the brain sections, although whether or not these substrates were limited to synapses is unclear. SDS-PAGE and autoradiography revealed numerous protein targets of [^3^H]AziP*m*. *Selectivity* was evident in that binding of [^3^H]AziP*m* did not correlate with protein abundance, which was estimated by Coomassie intensity (Fig. 2A). We also observed binding *specificity*, with inhibition of [^3^H]AziP*m* photolabeling of protein by only 100-fold higher concentrations of propofol (Fig. 2A). Based on optical density measurements of the entire lanes, 400 μM propofol decreased 4 μM [^3^H]AziP*m* binding to synaptosomal protein an average of 31%, with a maximum decrease of 57% in any individual band (Fig. 2A).

The autoradiograph signal normalized to the Coomassie intensity throughout the lanes revealed that, per amount of protein, higher molecular weight proteins were considerably more photolabeled than those of lower molecular weight (Fig. 2B). It seems unlikely that this is due to an artificially low Coomassie signal at higher molecular weights that could be caused by the absence of residues that favorably interact with the dye^12^.

Using protein standards, molecular weight could reliably be estimated between 10–250 kDa. In the absence of competing propofol, [^3^H]AziP*m* photolabeled 115–250 kDa protein proportionally with a small dependence on protein molecular weight (Fig. 2C). Less than 115 kDa, however, this trend was intermittently disrupted, presumably by abundant proteins without specific binding sites. Our interpretation is that longer polypeptides, because of larger surface area and more folds^13^, are statistically more likely to contain the structural features that constitute a specific binding site for the small alkylphenol anesthetics, whereas these features become progressively less likely as the proteins become smaller. A similar dependence of chain length on the creation of specific halothane binding sites has previously been demonstrated with model polypeptides^14^.

In the brain sections, high affinity binding to few proteins could have manifested as a highly selective drug distribution that mimics the expression of those targets. For example, if alkylphenols uniquely bound GABA_A_ receptors, a presumed functional target for these drugs, we would have expected greatest AziP*m* photolabeling in the cerebellar granule layer where functional GABA_A_ receptors are most abundant^15^,^16^. This would also have manifested as peak [^3^H]AziP*m* intensity only at ~60 kDa on the SDS-PAGE autoradiograph. However, the brain sections and SDS-PAGE gel together demonstrate the presence of many proteins that selectively and specifically bind alkylphenol general anesthetics. Identification of these many photolabeled proteins from synaptosomes will require purification of photolabeled protein or comprehensive protein sequencing to detect covalent AziP*m* adducts. Our previous attempts at identifying neuronal substrates of [^3^H]AziP*m* after photolabeling and IEF/SDS-PAGE provided evidence for strong binding primarily to mitochondrial β-barrels^10^,^17^. Together with our current data, this suggests that most proteins that bind alkylphenol anesthetics are difficult to solubilize and purify (e.g., helical transmembrane proteins), and thereby achieving the high sequence coverage with mass spectrometry that is necessary to detect adducts will be challenging.

### Lipid Photolabeling

To characterize alkylphenol anesthetic binding to rat brain lipids, we isolated major lipid species after photolabeling brain homogenate with 1 μM [^3^H]AziP*m*. Our thin layer chromatography and HPLC procedures enabled isolation of phosphatidylethanolamines (PE), phosphatidylcholines (PC), and cholesterol, as well as a fourth fraction that contained a mixture of phosphatidylserines (PS), phosphatidylinositols (PI), and sphingomyelins (SPH)^18^. [^3^H]AziP*m* exhibited statistically significant selectivity for photolabeling PE as compared to the other lipids (Table 3). [^3^H]AziP*m* binding to PE also increased in the presence of 400 μM propofol, in contrast to the other lipids, which showed no change in binding (PC and cholesterol) or a 50% decrease (PS/PI/SPH combined). Because of the abundance of PE, it seems clear that the inability of propofol to displace [^3^H]AziP*m* binding from brain slices was likely due to this over-abundance of non-specific sites, and that competition of labeling from proteins in brain sections was compensated for by this cooperative effect in the lipid fraction.

PE and PC, the dominant phospholipid species in the brain, are similarly zwitterionic, but differ in their polar headgroup compositions; the headgroup of PE contains an aminoethyl while that of PC contains a choline. Because the alkylphenols distribute near the headgroup of phospholipids at the hydrophobic-hydrophilic interface of bilayers^19^,^20^, it is possible that the selectivity of [^3^H]AziP*m* for binding PE compared to PC is due to the differences between these chemical groups. For example, the phenol hydroxyl of AziP*m* or propofol might better hydrogen bond to the less bulky headgroup of PE compared to PC. In separate experiments, we determined that 16 ± 3% of [^3^H]AziP*m* that covalently bound to PE had incorporated into the polar headgroup, as compared to only 5 ± 1% for both PC and PS/PI/SPH. Thus, at least in the absence of propofol, alkylphenols partially distribute to different membrane depths that are dependent upon adjacent phospholipid species. The cooperativity of AziP*m* photolabeling of PE in the presence of excess propofol might arise from displacement of [^3^H]AziP*m* from protein sites, or through alterations in bilayer structure or dynamics^19^,^21^,^22^ that permit greater access of the photolabel to these headgroup regions. The implications of this complex shifting of ligands between macromolecular pools are not clear, but emphasize that ligand-ligand interactions and the concentration-dependence of ligand actions are more complex than conventionally modeled.

### Conclusions

By photolabeling with the alkylphenol general anesthetic AziP*m*, we determined that neuronal binding is widespread and that the amount of anesthetic present in any macroscopic brain region is within about a two-fold range at equilibrium. Ligand distribution in the brain *ex vivo* is not dictated by few high affinity substrates that have restricted distribution. Alkylphenol anesthetic binding to protein is generally specific (i.e., saturable); however, in contrast to protein, alkylphenol anesthetic binding to lipid is both competitive and cooperative depending on the lipid species. Also, while AziP*m* has already proven to be a reliable mimic of propofol for binding to selected protein targets^3^,^17^,^23^,^24^,^25^, this work suggests broader applicability for AziP*m* in examining alkylphenol-protein interactions. Alkylphenol anesthetic protein substrates are widespread in both their number and distribution, similar to volatile general anesthetics, suggesting a multi-target pharmacologic mechanism for the on-pathway effects of general anesthetics, as well as a strong likelihood of multiple off-pathway targets.

## Methods

### Materials

Propofol (2,6-diisopropylphenol) was purchased from Sigma-Aldrich (St. Louis, MO), and AziP*m* was synthesized by published methods^3^. AziP*m* was tritiated by AmBios Labs (Boston, MA) and its application has been reported previously^10^,^17^,^23^,^24^,^26^. All protein assays were performed with a BCA protein assay kit from Thermo Fisher Scientific (Waltham, MA). For photolabeling, a Rayonet RPR-3500 lamp (Southern New England Ultraviolet Company, Branford, CT) with a 350 nm bulb was used^3^. Polyacrylamide gels (4–15%), PVDF membrane, and electrophoresis apparatuses were from Bio-Rad (Hercules, CA). Glass-backed thin layer chromatography plates were from Whatman (GE Healthcare, Little Chalfont, UK), and these 20 cm plates were coated with a 250 μm silica gel solid phase of 60 Å porosity. Purchased lipids were PC from egg yolk (Sigma-Aldrich), PS from porcine brain (Avanti Polar Lipids, Alabaster, AL), 1,2-dioleoyl-*sn*-glycero-3-phosphoethanolamine (DOPE) (Avanti Polar Lipids), and cholesterol (Sigma-Aldrich). For scintillation counting, EcoLite(+) liquid scintillation mixture (MP Biomedicals, Santa Ana, CA) was used with a PerkinElmer Life Sciences Tri-Carb 2800TR instrument (PerkinElmer, Waltham, MA). [^3^H]-sensitive film was from GE HealthCare, and developed films were scanned with a Bio-Rad GS-800 calibrated densitometer. All quantification from autoradiography films was performed using Quantity One (version 4.6.3) software.

### Animals

The animal protocol was approved as required by the University of Pennsylvania Institutional Animal Care and Use Committee, and all rats were treated in strict accordance to APS/NIH guidelines. A total of 5 adult female Sprague-Dawley rats (~300 g) that were purchased from Charles River Laboratories (Malvern, PA) were used for this study.

### Tissue preparation for section photolabeling

Rats were briefly anesthetized with halothane before perfusion with ice cold PBS, pH 7.4, via the left ventricle of the heart. The brains were quickly removed and cut into hemispheres with a sterile razor blade. The hemispheres were frozen in stirred isopentane cooled on dry ice, then stored at −80 °C. The brains were mounted in Tissue Tek O.C.T. compound (Sakura Finetek USA, Inc., Torrance, CA) and cut into 12 μm sagittal sections on a cryostat at −21 °C. Sections were mounted on chromium potassium sulfate and gelatin subbed glass slides, and the slides were stored at −80 °C until photolabeling.

### Brain section photolabeling

Slides were thawed to room temperature and rinsed in PBS to remove residual O.C.T. compound and any remaining halothane. The tissue sections were photolabeled in custom, gas-tight quartz cuvettes with 1 mm path length. The cuvettes contained 2.4 ml of 0.1 μM [^3^H]AziP*m* in PBS for binding distribution experiments with or without propofol (3–300 μM) for competition experiments. The cuvettes were equilibrated for 15 minutes in the dark then exposed to a 350 nm lamp for 15 minutes. The slides were then rinsed for 5 minutes in PBS before consecutive washes for 20 minutes each with fresh PBS, two washes with PBS containing 1 mg/ml bovine serum albumin, two washes with PBS, and one wash with distilled water. These washes removed unbound photolabel and salt. In pilot studies, these washes effectively removed detectable radioactive ligand from slides incubated with 0.1 μM [^3^H]AziP*m* without lamp exposure, tested by prolonged (60 day) exposure on autoradiography film and scintillation counting. Sections were dried over desiccant then placed on autoradiography film for 18 days. After development, the films were scanned with the densitometer, and the mean optical density of each region was quantified after subtracting background.

### Synaptosome isolation

Rats were briefly anesthetized, decapitated, and the brains were removed. The brains were briefly washed in isolation buffer (0.32 M sucrose and 5 mM Tris, pH 7.6, supplemented with protease and phosphatase inhibitors) then transferred to fresh isolation buffer. Brains were minced and homogenized by hand with a Teflon/glass Potter-Elvehjem tissue grinder. Synaptosomes were prepared essentially as described without detergent^27^. Purified synaptosomes were washed three times to remove residual Percoll by pelleting and resuspending in excess isolation buffer. Synaptosomes were resuspended in isolation buffer and stored at −80 °C.

### Synaptosome photolabeling, SDS-PAGE, and autoradiography

Synaptosomes corresponding to 200 μg of protein, as determined with protein assay, were diluted to 1 mg/ml in isolation buffer. 4 μM [^3^H]AziP*m* was added with 400 μM propofol or the DMSO vehicle for the control; the final DMSO concentration was 0.5%. After briefly vortexing and 3 minutes incubation on ice, the samples were photolabeled for 20 minutes in a quartz cuvette (pathlength 1 mm). The samples were then placed in a clean tube and pelleted at 14000 × g, and the pellet was washed twice with 800 μl 25 mM Tris, pH 7.6. After pelleting, the supernatant was discarded and the pellet was dissolved in 5% glycerol, 1% Triton X-100, 0.5% SDS, and 20 mM Tris, pH 7.6. The insoluble pellet was removed by centrifugation, and after protein assay, 50 μg of each sample was separated via SDS-PAGE. After SDS-PAGE, photolabeled protein was transferred to PVDF and exposed to film for 31 days. After development of the film, the membrane was stained with Commassie R-250, and both the film and membrane were scanned with the Bio-Rad GS-800 densitometer. Lane optical density was quantified from the film after subtracting background.

### Preparation of brain homogenate

Rats were anesthetized, decapitated, and the brains were removed as described above. The brains were washed in isolation buffer then transferred to fresh buffer for mincing and homogenizing. After homogenization, the homogenate was centrifuged at 1000 × g for 10 minutes, the pellet was discarded, and the supernatant was re-centrifuged at 1000 × g. After discarding the pellet, the supernatant was centrifuged at 13000 × g for 10 minutes. The supernatant was discarded, and the pellet was resuspended in isolation buffer. This brain homogenate fraction is devoid of most nuclei, connective tissue, and red blood cells, and was used for experiments characterizing lipid photolabeling.

### Photolabeling of brain homogenate and lipid isolation

Brain homogenate was diluted to 1.25 mg protein/ml in isolation buffer. For each lipid isolation experiment, brain homogenate corresponding to 1.5 mg protein was photolabeled for 20 minutes with 1 μM [^3^H]AziP*m* in the presence of 400 μM propofol or the DMSO vehicle for the control; the final DMSO concentration was 0.25%. After photolabeling, the homogenate was centrifuged for 10 minutes at 14000 × g. The supernatant was discarded, and the pellet was resuspended to 1 mg/ml in 5 mM Tris, pH 7.4. This was re-centrifuged at 14000 × g, the supernatant was discarded, and the pellet was resuspended to 10 mg/ml in 2 mM Tris, pH 7.4. From this, lipids were isolated with a Folch extraction^28^. Briefly, after transfer to a glass vial, chloroform and methanol were added to achieve a final ratio of 8:4:3 chloroform:methanol:H_2_O. After thorough mixing, the samples were centrifuged at 1000 × g for five minutes, then the lipid-containing organic layer was isolated.

### Thin layer chromatography and plate analysis

Lipid samples were concentrated to ~150 μl under N_2_ gas before spotting on silica gel plates. For use as standards, the following were dissolved in methanol and were spotted in individual lanes adjacent to the photolabeled samples: 250 μg PC, 250 μg PS, 250 μg DOPE, 250 μg cholesterol, and 500 nmol AziP*m*. Passive separation was achieved with a mobile phase of 64:25:4 chloroform:methanol:(28.5%) ammonium hydroxide until the migrating front was ~1 cm from the top of the plates.

After separation, the plates were dried overnight then stained in a glass box with iodine vapor. Lipid spots were marked in pencil, and the plates were scanned. The scanned plates were used to determine spot Rf (retardation factor), which was measured from the center of the spots. The spots were scraped off the silica plates into scintillation vials for lipid elution and subsequent analyses.

### Phospholipid scintillation counting and phosphorous assay

Phospholipids were eluted from the scraped silica spots by adding 1 ml of 1:1 chloroform:methanol into the scintillation vials and incubating overnight with gentle agitation. 100 μl of the eluates were added to separate scintillation vials for scintillation counting.

The remaining 900 μl (excluding the silica) were placed in glass test tubes for measuring total phospholipid via a phosphorous assay^29^. The solvent was evaporated with N_2_, and 450 μl of 8.9 N H_2_SO_4_ was added to each tube. This was heated to > 200 °C for 40 minutes or until the lipid was dark yellow. This was then cooled to room temperature before 150 μl of 30% H_2_O_2_ was added. This was heated for 30 minutes or until clear, then cooled to room temperature. 3.9 ml of H_2_O and 500 μl of 2.5% ammonium molybdate tetrahydrate were added to each tube before mixing. 500 μl of 10% ascorbic acid was then added, and the samples were mixed then incubated at room temperature for 30 minutes. Absorbance at 820 nm was measured with a Varian Cary 300 Bio UV-visible spectrophotometer (Varian, Inc., Palo Alto, CA). Parallel with processing samples, a standard curve was generated with known amounts (0 to 0.65 μmol) of PC, and a linear (R^2^ = 0.99) relationship between absorbance and total phosphorous was reproducible.

### Phospholipid hydrolysis and scintillation counting

After elution of phospholipids from scraped silica, samples were dried with N_2_ and resuspended in 1 ml of 9:1 acetonitrile:0.5 N HCl. This was heated to 100 °C for forty-five minutes, then 3.2 ml of 5:1 chloroform:H_2_O was added. This was centrifuged at 3000 × g for five minutes, and the organic layer (containing acyl chains) and the aqueous layer (containing polar headgroups) were isolated separately^30^. An additional 0.5 ml of H_2_O was added to the organic layer for a second extraction, and after centrifugation, the aqueous fractions were combined. 100 μl each of the organic and aqueous fractions were scintillation counted, and relative cpm in each layer was determined after adjusting for the total extraction volumes.

### Cholesterol scintillation counting and cholesterol assay

Similar to phospholipids, cholesterol was eluted from the scraped silica into 1 ml of 1:1 chloroform:methanol. Before quantifying [^3^H]AziP*m* binding to cholesterol, it was essential to first remove non-cholesterol bound [^3^H]AziP*m*, which migrated similar with cholesterol on chromatography plates. To achieve this, the eluted samples were dried with N_2_ then further dried overnight under a vacuum. To each sample, 150 μl of isopropanol was added, and the samples were incubated at room temperature for 2.5 hours with mild agitation. Samples were briefly sonicated for 60 seconds, then centrifuged at 14000 × g for 20 minutes. The supernatant was extracted, and an aliquot was used to determine the cholesterol concentration using a cholesterol quantitation kit (Sigma-Aldrich). A separate 15 μl of the supernatant was then separated by reverse phase-high performance liquid chromatography with a C18 analytical column. An isocratic mobile phase of 60:35:5:0.1 ACN:isopropanol:H_2_O:TFA with a 1 ml/min flow rate at room temperature was used, and analytes were detected using UV-Vis absorbance at 373 nm and 210 nm. Processed in parallel with brain homogenate, pure [^3^H]AziP*m* and cholesterol controls were analyzed and resolved peaks at 3.4 and 8.5 minutes, respectively. Within brain homogenate samples, the cholesterol peak remained clearly distinct from [^3^H]AziP*m* and unbound photolysis products, which eluted within the first 5 minutes. The cholesterol fraction, photolabeled and otherwise, was collected from 6–20 minutes and dried to 2 ml with N_2_. To this, scintillation fluid was added for counting.

### Statistics

Standard error is shown for mean values. Statistical analyses were performed with the GraphPad Prism (version 6.0e) software (GraphPad Software, Inc., La Jolla, CA). Detailed statistical procedures are described in the legends where applicable.

## Figures and Tables

**Table 1:** [^3^H]AziP*m* binding to rat brain regions

Brain region	0.1 μM [^3^H]AziP*m* (mO.D. ± SE)*a*	0.1 μM [^3^H]AziP*m* + 300 μM propofol (mO.D. ± SE)	AziP*m* selectivity ratio*b*	Halothane selectivity ratio*c*
Cortex	215 ± 9	199 ± 11	0.14	0.13
Corpus callosum	147 ± 11	151 ± 7	0.10	0.12
Hippocampal molecular layer	199 ± 12	190 ± 11	0.13	0.13
Hippocampal pyramidal layer	168 ± 7	157 ± 7	0.11	0.10
Dentate molecular layer	212 ± 12	198 ± 7	0.14	0.12
Dentate granule cell layer	145 ± 7	141 ± 6	0.10	0.07
Cerebellar molecular layer	182 ± 6	178 ± 6	0.12	0.14
Cerebellar granular layer	118 ± 5	125 ± 2	0.08	0.07
Cerebellar white matter	100 ± 7	97 ± 5	0.07	0.12

^a^Milli-optical density (mO.D.) data is from (n = 4) brain sections for [^3^H]AziP*m* and (n = 8) sections for [^3^H]AziP*m* + propofol.

^b^Selectivity ratio calculated as region mO.D./sum of mO.D. from all the regions.

^c^Data for halothane derived from Ref. 8.

**Table 2:** [^3^H]AziP*m* binding to rat brain by compositional region (mO.D. ± SE)

Brain region	0.1 μM [^3^H]AziP*m*
Molecular layers	202 ± 8*a*
Cortex	
Hippocampus	
Dentate gyrus	
Cerebellum	
Cell body layers	144 ± 15
Hippocampus pyramidal	
Dentate gyrus granule cell	
Cerebellar granular/Purkinje	
White matter	124 ± 24
Corpus callosum	
Cerebellum	

^a^Significantly greater binding in the molecular layers compared to cell body or white matter regions was determined with one-way ANOVA ( p = 0.01) followed by Bonferroni post-hoc tests comparing all means and testing for significance with a family-wise error rate of 0.05. Mean values represent averages from 0.1 μM [^3^H]AziP*m* binding in Table 1.

**Table 3:** [^3^H]AziP*m* binding to major rat brain lipids

Lipids	Rf value	pmol [^3^H]AziP*m* binding per μmol lipid	Fold change with 400 μM propofol co-incubation
PE	0.7 ± 0.1	102.1 ± 24.1*a*	1.7 ± 0.2
PC	0.4 ± 0.1	36.5 ± 10.6	0.9 ± 0.2
PS/SPH/PI	0.2 ± 0.1	27.0 ± 10.8	0.4 ± 0.1
Cholesterol	1	0.8 ± 0.1	1.2 ± 0.2

^a^Significantly greater [^3^H]AziP*m* binding to PE compared to PC, PS/SPH/PI, and cholesterol was determined with one-way ANOVA (p = 0.002) followed by Bonferroni post-hoc tests comparing all means and testing for significance with a family-wise error rate of 0.05. Mean values are from (n = 4) experiments per lipid. PE, phosphatidylethanolamines; PC, phosphatidylcholines; PS, phosphatidylserines; SPH, sphingomyelins; PI, phosphatidylinositols; Rf, retardation factor.
